# Rapid PCR-Based Nanopore Adaptive Sequencing Improves Sensitivity and Timeliness of Viral Clinical Detection and Genome Surveillance

**DOI:** 10.3389/fmicb.2022.929241

**Published:** 2022-06-16

**Authors:** Yanfeng Lin, Yan Dai, Yuqi Liu, Zhuli Ren, Hao Guo, Zhenzhong Li, Jinhui Li, Kaiying Wang, Lang Yang, Shuang Zhang, Hongbo Liu, Leili Jia, Ming Ni, Peng Li, Hongbin Song

**Affiliations:** ^1^Academy of Military Medical Sciences, Academy of Military Sciences, Beijing, China; ^2^Chinese PLA Center for Disease Control and Prevention, Beijing, China; ^3^State Key Laboratory of Translational Medicine and Innovative Drug Development, Jiangsu Simcere Diagnostics Co., Ltd., Nanjing, China; ^4^Changchun Veterinary Research Institute, Chinese Academy of Agricultural Sciences, Changchun, China; ^5^Nanjing Simcere Medical Laboratory Science Co., Ltd., Nanjing, China; ^6^Institute of Health Service and Transfusion Medicine, Beijing, China

**Keywords:** nanopore sequencing, adaptive sequencing, human adenovirus, SARS-CoV-2, pathogen detection

## Abstract

Nanopore sequencing has been widely used for the real-time detection and surveillance of pathogens with portable MinION. Nanopore adaptive sequencing can enrich on-target sequences without additional pretreatment. In this study, the performance of adaptive sequencing was evaluated for viral genome enrichment of clinical respiratory samples. Ligation-based nanopore adaptive sequencing (LNAS) and rapid PCR-based nanopore adaptive sequencing (RPNAS) workflows were performed to assess the effects of enrichment on nasopharyngeal swab samples from human adenovirus (HAdV) outbreaks. RPNAS was further applied for the enrichment of severe acute respiratory syndrome coronavirus 2 (SARS-CoV-2) from nasopharyngeal swab samples to evaluate sensitivity and timeliness. The RPNAS increased both the relative abundance (7.87–12.86-fold) and data yield (1.27–2.15-fold) of HAdV samples, whereas the LNAS increased only the relative abundance but had no obvious enrichment on the data yield. Compared with standard nanopore sequencing, RPNAS detected the SARS-CoV-2 reads from two low-abundance samples, increased the coverage of SARS-CoV-2 by 36.68–98.92%, and reduced the time to achieve the same coverage. Our study highlights the utility of RPNAS for virus enrichment directly from clinical samples, with more on-target data and a shorter sequencing time to recover viral genomes. These findings promise to improve the sensitivity and timeliness of rapid identification and genomic surveillance of infectious diseases.

## Introduction

Infectious diseases have posed a major challenge to public health for centuries (Morens et al., 2004). Rapid and accurate pathogen detection represents the primary step of disease prevention and control. Compared with traditional next-generation sequencing (NGS), nanopore sequencing has the advantages of real-time, long read length, and portability, making it suitable for rapid pathogen detection (Lewandowski et al., 2019). Recently, nanopore sequencing has been widely used in the field of outbreak investigations and genome surveillance [e.g., Ebola (Quick et al., 2016), Zika (Faria et al., 2016), and SARS-CoV-2 (Meredith et al., 2020)], as well as clinical infections (Gu et al., 2021). However, the high host background and low microbial content limited the application of nanopore sequencing of the clinical samples (Greninger et al., 2015). The low throughput of nanopore sequencing reduces the sensitivity of pathogen detection, making it more difficult for pathogen traceability and typing. The enrichment of target pathogens is particularly important for nanopore sequencing, and thus, a series of enrichment methods have been developed. Saponin-based removal of host nucleic acid can achieve a median 600-fold depletion of human DNA from respiratory samples, but it is only applicable to bacteria with intact cell walls (Charalampous et al., 2019). The probe hybridization capture approach (Schuele et al., 2020) and multiplex PCR amplification (Quick et al., 2017) have been applied to viral enrichment but have been restricted to certain known types of pathogens. Moreover, these methods require complicated pretreatment of the samples, which cannot meet the demands of point-of-care sequencing.

Oxford Nanopore Technologies recently launched an adaptive sequencing function by aligning the reads to the references and ejecting uninterested reads by reversing the voltage across individually selected nanopores in real-time, which could achieve a computational enrichment of on-target sequences without an additional pretreatment process (Loose et al., 2016; Bao et al., 2021). Previous studies have developed adaptive sequencing tools using a graphical processing unit base-calling (readfish) (Payne et al., 2021) or raw electrical signal mapping (UNCALLED) (Kovaka et al., 2021). Sequencing enrichment of the human genome, tumor genes, or specific species in a mock microbial community has been observed by adaptive sequencing. For human genetic diagnosis, adaptive sequencing has been used to accurately identify pathogenic structural variants (Miller et al., 2021; Stevanovski et al., 2022) and determine the structure of the small supernumerary marker chromosomes (Mariya et al., 2022). Combined with the enzymatic removal of the host background, adaptive sequencing obtained a 113.41-fold enrichment of microbial DNA in respiratory tract samples (Gan et al., 2021). Through the direct depletion of host DNA using adaptive sequencing, Marquet et al. (2022) achieved a 1.70-fold (±0.27-fold) enrichment without changing the microbial composition ratio of the samples. However, the application of adaptive sequencing for the rapid pathogen detection of infectious disease outbreaks remains lacking and requires further evaluation.

In the present study, we first performed adaptive sequencing of samples from a human adenovirus (HAdV) outbreak and evaluated the enrichment efficiency of two different workflows. The rapid PCR-based nanopore adaptive sequencing (RPNAS) workflow, which showed a better enrichment effect than the ligation-based nanopore adaptive sequencing (LNAS) workflow, was further applied to enrich severe acute respiratory syndrome coronavirus 2 (SARS-CoV-2) from respiratory samples. Our study aimed to evaluate the utility of adaptive sequencing for virus detection and genome enrichment from clinical samples.

## Materials and Methods

### Sample Collection, Nucleic Acid Extraction, and Real-Time PCR Assay

The nasopharyngeal swab specimens used in this study were from the 2019 outbreak of HAdV in Hubei province, China. The nucleic acid of 10 HAdV-positive specimens was extracted and the Ct values were determined by real-time PCR (RT-PCR) in our previous study (Li et al., 2021). A total of 10 nasopharyngeal swab specimens from case-patients with COVID-19 were collected in January 2021 through routine surveillance. A 200 μl sample of each specimen was used for nucleic acid extraction using a LabServ Prefill Viral Total NA Kit (Fisher Scientific, Waltham, MA, United States) following the manufacturer’s instructions. A commercial RT-PCR assay kit (BioGerm, Shanghai, China) was used to determine the SARS-CoV-2 Ct values by targeting ORF1ab and N genes, with the human RNaseP gene used as a control. All of the clinical samples used in this study were collected through routine surveillance, as no personally identifiable data were included. The ethics of the study were reviewed and supervised by the Chinese PLA Center for Disease Control and Prevention.

### Nanopore Library Preparation and Sequencing of the Clinical Samples

The remnant nucleic acid of HAdV samples was used for adaptive sequencing. Two nanopore adaptive sequencing workflows were used in this study (Supplementary Figure 1). Samples M9 and HB204 with higher DNA concentrations were first selected for pilot evaluation. For the LNAS workflow, approximately 10–30 ng DNA was used for library preparation with a Ligation Sequencing Kit (Oxford Nanopore Technologies, Cambridge, United Kingdom). Barcodes were added with a Native Barcoding Expansion kit for each sample. The adaptor-ligated library was cleaned up with 0.8× AMPure XP beads (Beckman Coulter, Indianapolis, IN, United States). For the RPNAS workflow, approximately 3 ng DNA was used for library preparation with a Rapid PCR Barcoding Kit (Oxford Nanopore Technologies, Cambridge, United Kingdom) with the number of PCR cycles set at 25.

The prepared libraries were sequenced on a MinION with an R9.4.1 flow cell (FLO-MIN106). A desktop computer with an Intel Core i7-10700F CPU and NVIDIA GeForce RTX 2070 SUPER GPU was used, and adaptive sequencing was performed using MinKNOW (v21.05.20) with a fast-basecalling model and set as an enrichment mode with the whole HAdV-55 genome (Genbank accession number: MT806175.1) used as the reference. The channels in the flow cell were separated into two groups: channel 1–256 was set as the enriched group (adaptive sequencing) and channel 257–512 as the control group (standard sequencing). Reads were mapped to the reference genome of HAdV-55 using an in-built minimap2 (Li, 2018).

For the SARS-CoV-2 samples, reverse transcription and second-strand cDNA synthesis were performed prior to library preparation using the NEBNext Ultra II RNA First-Strand Synthesis Module and the NEBNext Ultra II Non-directional RNA Second Strand Synthesis Module (New England BioLabs, Ipswich, MA, United States) with both random hexamers and oligo-dT primers. Synthesis products were cleaned using a 1:1 ratio of AMPure XP beads and quantified using the Qubit dsDNA HS kit (Thermo Fisher Scientific, Waltham, MA, United States). The samples were sequenced with the RPNAS workflow as described above using the SARS-CoV-2 reference genome (Genbank accession number: MN908947.3).

### Bioinformatics and Statistical Analysis

For the assessment of adaptive sequencing performance, the log files for adaptive sequencing were used to discriminate the signal sent to the pore for each read in the enriched group, which included three decisions: the reserved target reads (stop receiving), the ejected non-target reads (unblock), and the reads that were aligned but could not be classified (no decision). In addition, reads generated by channels corresponding to the enriched group but not recorded in the log file were defined as non-adaptive reads. The classification of decisions was then merged with more read details, such as read-id, sequencing time, read length, and channel location in the sequencing-summary files, followed by a comparison between the enriched and control groups. The read length distributions of different decisions were visualized using a violin plot, and *p*-values were calculated with Student’s *t*-test. Channels in which sequencing occurred between the intervals of adjacent time were defined as active. BAM files were processed to calculate the depth and coverage using minimap2 v2.21 and SAMtools v1.13 (Li et al., 2009) with default parameters. Statistics of the total yield, mean depth, coverage, and active channels over time were calculated in sliding windows for every 15 min. A comparison of mean depth between two groups was performed using the Wilcoxon signed-rank test. All plots and tables were created in R 4.1.2.

## Results

### Performance of Adaptive Sequencing With the Ligation-Based Nanopore Adaptive Sequencing and Rapid PCR-Based Nanopore Adaptive Sequencing Workflows

**FIGURE 1:**
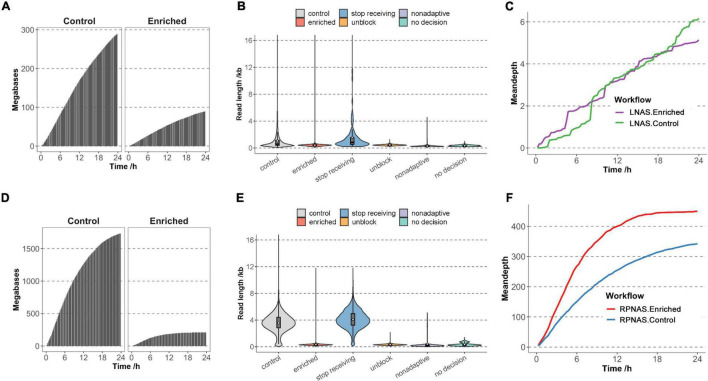
Adaptive sequencing performance of the ligation-based nanopore adaptive sequencing (LNAS) and rapid PCR-based nanopore adaptive sequencing (RPNAS) workflows. The cumulative total base yield in the enriched and control groups over 24 h sequencing of sample M9 with the LNAS workflow **(A)** and sample HB204 with the RPNAS workflow **(D)**. The read length distribution of sample M9 with the LNAS workflow **(B)** and sample HB204 with the RPNAS workflow **(E)**. Reads in both the enriched and control groups were represented with red and gray colors, separately. Blue, orange, purple, and green colors represent stop receiving, unblock, non-adaptive, and no decision, respectively. The white dot represents the mean read length of each decision. The mean depth of human adenovirus (HAdV) in both the enriched and control groups over 24 h sequencing of sample M9 with LNAS workflow **(C)** and sample HB204 with RPNAS workflow **(F)**.

To evaluate the effect of different workflows on adaptive sequencing, we first used sample M9 from an HAdV outbreak to enrich HAdV genomes with the LNAS workflow. Channels on the flow cell were divided into the enriched group with adaptive sequencing (channel 1–256) and the control group with standard sequencing (channel 257–512). In the 24 h sequencing of M9, MinION generated 381 MB of data with 489,633 reads. The enriched group obtained fewer data and a lower increase rate of the bases than that of the control group (9.00 × 10^7^ vs. 2.91 × 10^8^ bases) (Figure 1A and Supplementary Table 1). The median read length of the enriched and control groups was 417 and 596 bp (*p* < 0.001), respectively, and most of the reads were shorter than 1 kb. In the enriched group, 78.42% of reads were identified as unblocked reads with a median length of 441 bp. Non-adaptive and no decision reads constituted 20.44% and 1.09%, respectively, with shorter fragments (median length of 278 and 346 bp, respectively), and only 0.05% reads were identified as stop receiving reads with a median length of 840 bp (Figure 1B). A total of 142 reads were mapped to HAdV in the enriched group, whereas there were 156 reads in the control group. However, adaptive sequencing obtained an increased relative abundance of HAdV bases (0.21% in the enriched group and 0.08% in the control group). There was no promotion of the total HAdV base yield (1.90 × 10^5^ bases in the enriched group and 2.28 × 10^5^ bases in the control group). The mean depth of HAdV in the enriched group was slightly higher than that in the control group during the early stages of sequencing (approximately 8 h); however, no significant differences were observed after approximately 10 h of sequencing (Figure 1C). In addition, adaptive sequencing generated additional HAdV reads in the enriched group than that in the control group in the first 12 h, which suggested the potential for rapid pathogen detection (Supplementary Figure 2).

Sample HB204 was further used to evaluate the enrichment performance of the RPNAS workflow, which generated 1.09 × 10^6^ reads with 1.95 × 10^9^ bases in 24 h. Adaptive sequencing generated more reads in the enriched group (6.08 × 10^5^ reads) than that in the control group (4.85 × 10^5^ reads), but the total bases in the enriched group were much lower than that of the control group (2.11 × 10^8^ vs. 1.74 × 10^9^ bases) (Figure 1D and Supplementary Table 1). The median read length of the enriched group (307 bp) was much shorter than that of the control group (3,607 bp) (*p* < 0.001) due to the ejection of unwanted sequences, which lead to additional reads with a short length (Figure 1E).

In 24 h of sequencing, 4,397 reads (1.72 × 10^6^ bases) and 3,411 reads (1.35 × 10^6^ bases) were identified as HAdV in the enriched and control groups, respectively, which showed both the enrichment of relative abundance (10.48-fold) and data yield (1.27-fold) of HAdV. Moreover, the mean depth of HAdV in the enriched group was significantly higher than that in the control group through all timestamps (*p* < 0.05). However, the increasing rate of the mean depth in the enriched group decreased rapidly along the sequencing time (Figure 1F).

### Validation of the Enrichment Effect of Nanopore Adaptive Sequencing With Nine Human Adenovirus Samples

**FIGURE 2:**
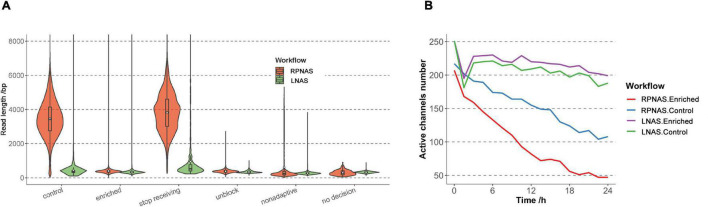
Sequencing performance of the RPNAS and LNAS workflows with pooled samples. **(A)** Read length distributions with different decisions in two workflows. Reads in RPNAS and LNAS workflow were indicated in red and green, respectively. **(B)** Changes in the active channel numbers over 24 h of sequencing in different workflows.

The above results showed that the RPNAS workflow had a better enrichment effect on adaptive sequencing than the LNAS workflow. To verify the repeatability, another nine samples with different Ct values were pooled and sequenced by the LNAS and RPNAS workflow on two flow cells, which generated 3.58 and 2.70 M reads within 24 h, respectively (Supplementary Table 1). The median read length of the control group in the RPNAS workflow (3,415 bp) was much higher than that in the LNAS workflow (346 bp) (*p* < 0.001), whereas the read length of the enriched groups in both workflows was short (median read length < 400 bp), which was consistent with the above results in a single sample (Figure 2A). Furthermore, channel attrition rates were estimated by calculating changes in the active channel numbers, and the channel attrition rates of the enriched group were similar to that of the control group in the LNAS workflow but higher than the control group in the RPNAS workflow (Figure 2B).

**FIGURE 3:**
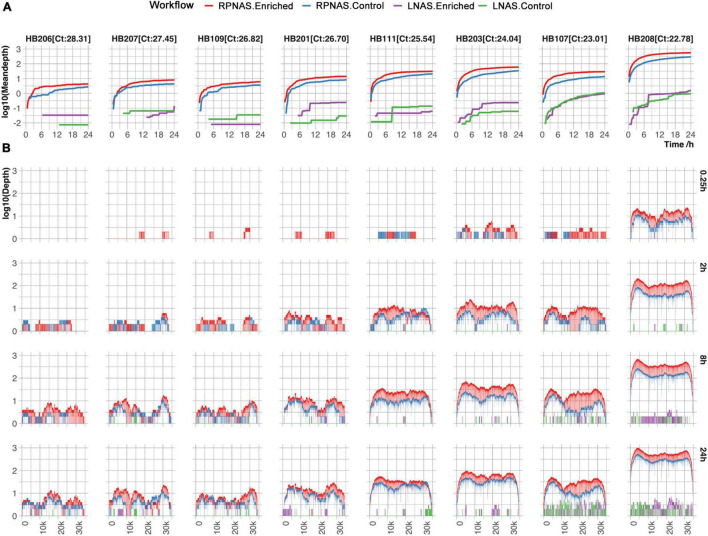
Comparison of HAdV enrichment in eight samples with different workflows. **(A)** The mean depth of the HAdV genomes changed over 24 h of sequencing. **(B)** Coverage of HAdV genomes at the different timestamps (0.25, 2, 8, and 24 h). Red, blue, purple, and green lines represent the enriched group in RPNAS workflow, the control group in RPNAS workflow, the enriched group in LNAS workflow, and the control group in LNAS workflow, respectively.

Subsequently, the HAdV reads from each sample were analyzed. Sample HB205 was not included, with only one read detected in both the LNAS and RPNAS workflows. The mean depth in the enriched group increased faster than that of the control group in the RPNAS workflow, and the RPNAS workflow had a much higher mean depth of HAdV than the LNAS workflow (Figure 3A). Comparing the coverage of the HAdV genome at each of the different timestamps (0.25, 2, 8, and 24 h), all samples gradually approached 100% coverage over the reference, and a higher depth was observed in samples with lower Ct values for the same timestamp. A higher coverage was always observed in the enriched group in the RPNAS workflow (Figure 3B). The RPNAS workflow obtained a 1.50–2.15-fold enrichment of HAdV data yield and a 7.87–12.86-fold enrichment of HAdV relative abundance (Supplementary Table 2). However, inconspicuous enrichment was observed in the LNAS workflow with 0.24–7.08-fold enrichment of the HAdV data yield and 0.22–7.87-fold enrichment of HAdV relative abundance (Supplementary Table 2).

### Enrichment of SARS-CoV-2 Genomes From Nasopharyngeal Swab Samples by Adaptive Sequencing

**FIGURE 4:**
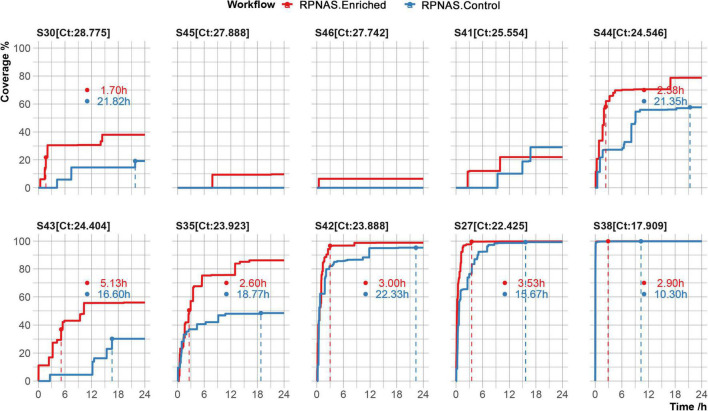
Genome coverage of the severe acute respiratory syndrome coronavirus 2 (SARS-CoV-2) reference over 24 h in 10 samples. The time to reach the max coverage of the control group is indicated by the dotted lines.

To evaluate the potential utility of adaptive sequencing on viral sequencing, 10 SARS-CoV-2 positive samples were collected and the viral genome was enriched using the RPNAS workflow. Nanopore sequencing generated 3.9 × 10^5^–1.1 × 10^6^ total reads per sample. In all samples, SARS-CoV-2 reads were detected in a 24 h sequencing period. Adaptive sequencing detected SARS-CoV-2 in samples S46 and S45 with 1 and 2 reads from the enriched group, whereas no SARS-CoV-2 read was detected in the control group. In sample S41, the first SARS-CoV-2 read (1 h 58 min) was detected much earlier in the enriched group compared with the control group (9 h 22 min). This finding indicated that adaptive sequencing could detect pathogens with greater sensitivity from low-abundance samples compared with standard metagenomic sequencing. The other seven samples generated 10 to 37,733 SARS-CoV-2 reads, and the enriched group obtained 1.52–2.90-fold enrichment in the data yield and 4.02–9.35-fold enrichment in the relative abundance of SARS-CoV-2 compared with the control group (Supplementary Table 3). We further calculated the time taken to reach the maximum coverage of each sample. In samples S30, S44, S43, and S35, we observed that SARS-CoV-2 coverage in the enriched group was increased by 36.68–98.92% compared with the control group after 24 h. In samples S42, S27, and S38, the coverage of SARS-CoV-2 exceeded 95% in both the enriched and control groups. However, the enriched group took less time to achieve the same coverage of SARS-CoV-2 compared with the control group (3.14 vs. 16.10 h on average) (Figure 4).

## Discussion

In the present study, we evaluated the utility of nanopore adaptive sequencing to enrich the viral genome directly from respiratory samples. First, we performed adaptive sequencing on HAdV positive samples to evaluate the enrichment performance of two different workflows. The LNAS workflow increased the relative abundance of HAdV, indicating a certain enrichment effect. However, adaptive sequencing only slightly increased the mean depth in the enriched group during the early stage and failed to improve the data yield. The RPNAS workflow increased both the relative abundance and data yield of HAdV. The effective enrichment of SARS-CoV-2 suggested that RPNAS could achieve rapid pathogen detection with higher sensitivity and reduce the sequencing time to recover the genome sequences of the pathogen compared with standard nanopore sequencing.

The length of DNA fragments from the samples is an important factor affecting the enrichment effect of adaptive sequencing, and libraries with longer fragment lengths showed better enrichment performance (Zhao et al., 2021; Martin et al., 2022). In this study, the RPNAS workflow, which generated a longer length of library fragments, also showed better enrichment performance than that of the LNAS workflow. This finding was likely due to long fragment PCR amplification, and size selection with magnetic beads increased the proportion of longer DNA fragments. In contrast, the low concentration of the sequencing library and short DNA fragments in the LNAS workflow may lead to inconspicuous enrichment. For RNA viruses (e.g., SARS-CoV-2), it is challenging to synthesize full-length cDNA before sequencing, which is critical for enrichment performance. Therefore, maintaining the integrity and avoiding nucleic acid degradation during sample processing may improve the enrichment effects of adaptive sequencing. Extraction of high molecular weight DNA can help obtain longer DNA fragments as much as possible, to improve the enrichment performance of adaptive sequencing (Mayjonade et al., 2016; Angthong et al., 2020; Penouilh-Suzette et al., 2020; Boughattas et al., 2021; Jones et al., 2021). Previous studies have found that the loss of active channels was faster during adaptive sequencing (Payne et al., 2021), and read rejections lead to a reduction of the overall data throughput (Martin et al., 2022; Sun et al., 2022), which may decrease the enrichment effect of adaptive sequencing. We also observed a decline in the overall data yield during adaptive sequencing. Therefore, increasing the enrichment efficiency is a key factor for the application of adaptive sequencing, which requires further optimization. The existing adaptive sequencing methods are primarily based on the alignment of nucleotide sequences or raw electrical signals, which could achieve approximately a 5-fold maximum enrichment of target genome data (Gan et al., 2021; Kipp et al., 2021; Kovaka et al., 2021; Payne et al., 2021; Wanner et al., 2021; Martin et al., 2022). A deep-learning model distinguishes human DNA from bacterial DNA with over 90% accuracy and is faster than alignment-based approaches, which could represent an alternative option for adaptive sequencing to increase enrichment efficiency (Bao et al., 2021).

There were some limitations associated with this study. The amount and remaining DNA in the samples used in this study were limited. Sample M9 was exhausted during the LNAS workflow, and another sample HB204 was selected for an evaluation of the RPNAS workflow. Moreover, the pathogen in all of the samples was identified and the specific reference genomes of HAdV or SARS-CoV-2 could be selected in advance. However, it is often difficult to know which pathogen is present in the samples prior to detection and makes it impossible for pathogen enrichment. A panel of multi-reference genomes could be established for the detection and enrichment of different concerned pathogens. Meanwhile, depleting the reads of the host with the human genome as the reference is an alternative strategy, which can increase microbial data output without changing the microbial composition in the sample (Marquet et al., 2022).

In conclusion, our study highlighted the utility of RPNAS for the enrichment of viral genomes from clinical samples. Adaptive sequencing could obtain more targeted genome data and shorten the time to recover viral genomes, which holds promise as an application as a point-of-care assay for pathogen detection and surveillance of infectious diseases with higher sensitivity and shorter timeliness.

## Data Availability Statement

The datasets presented in this study can be found in online repositories. The names of the repository/repositories and accession number(s) can be found below: https://www.ncbi.nlm.nih.gov/, PRJNA816258.

## Ethics Statement

The studies involving human participants were reviewed and approved by the Chinese PLA Center for Disease Control and Prevention. Written informed consent for participation was not required for this study in accordance with the national legislation and the institutional requirements.

## Author Contributions

YFL, JL, and KW performed the experiment. YD, YQL, ZR, ZL, LY, and SZ performed the formal analysis. HL and LJ collected the samples. YFL, YD, and ZR wrote and revised the draft of the manuscript. HG, MN, PL, and HS designed the study and revised the manuscript. All authors contributed to the article and approved the submitted version.

## Conflict of Interest

YD, HG, and ZL were employed by Jiangsu Simcere Diagnostics Co., Ltd. HG was employed by Nanjing Simcere Medical Laboratory Science Co., Ltd. The remaining authors declare that the research was conducted in the absence of any commercial or financial relationships that could be construed as a potential conflict of interest.

## Publisher’s Note

All claims expressed in this article are solely those of the authors and do not necessarily represent those of their affiliated organizations, or those of the publisher, the editors and the reviewers. Any product that may be evaluated in this article, or claim that may be made by its manufacturer, is not guaranteed or endorsed by the publisher.
