# Supplementary figures and images for: Rapid PCR-Based Nanopore Adaptive Sequencing Improves Sensitivity and Timeliness of Viral Clinical Detection and Genome Surveillance
Source: Front Microbiol. 2022 Jun 16;13:929241. doi: 10.3389/fmicb.2022.929241 (PMC9244360; doi:10.3389/fmicb.2022.929241)

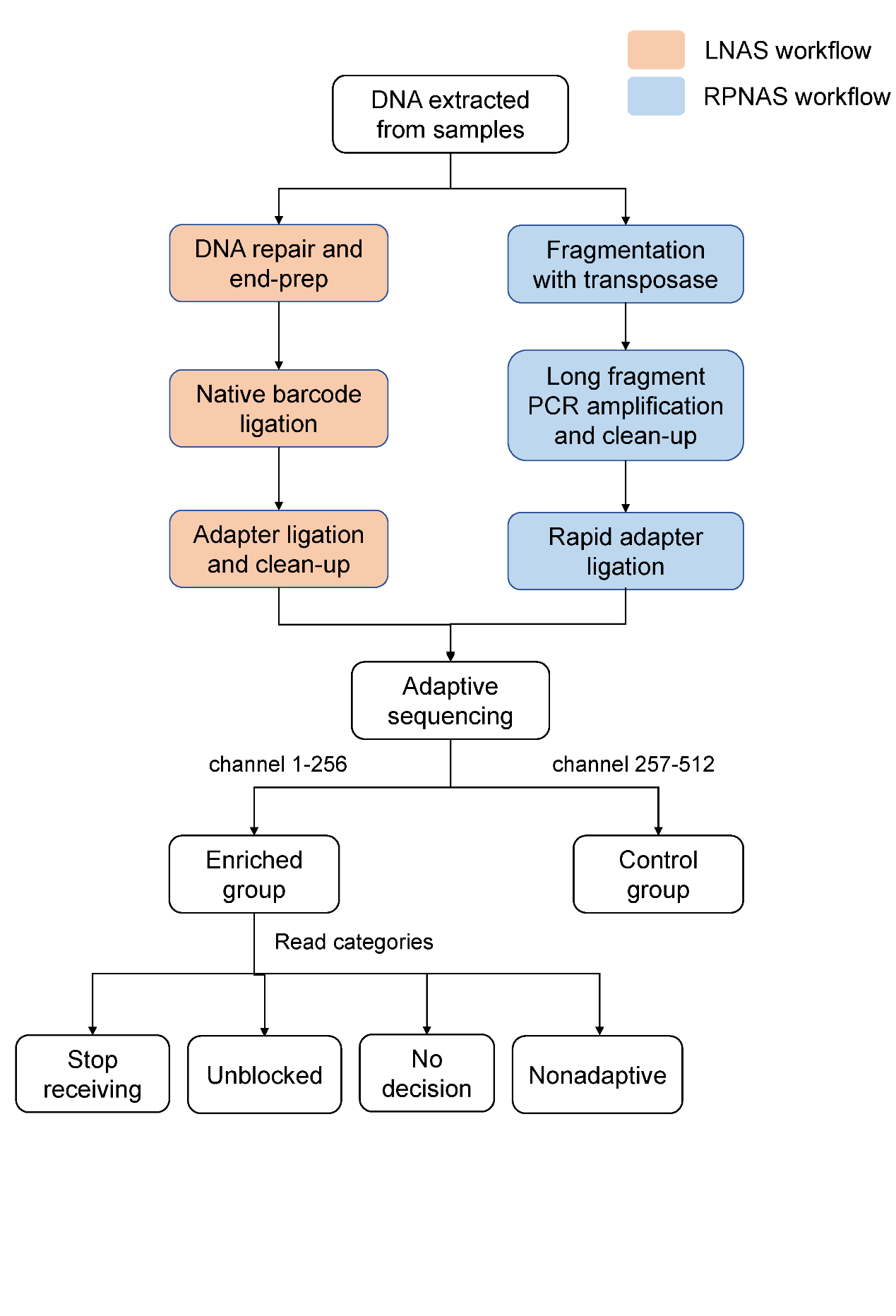
 **Figure S1. Overview of nanopore adaptive sequencing with LNAS and RPNAS workflow.**

Supplement: Supplementary file 1 [file Data_Sheet_1.DOCX]
