# Supplementary material for: Rapid PCR-Based Nanopore Adaptive Sequencing Improves Sensitivity and Timeliness of Viral Clinical Detection and Genome Surveillance
Source: Front Microbiol. 2022 Jun 16;13:929241. doi: 10.3389/fmicb.2022.929241 (PMC9244360; doi:10.3389/fmicb.2022.929241)
Supplement: Supplementary file 2 [file Data_Sheet_2.DOCX]

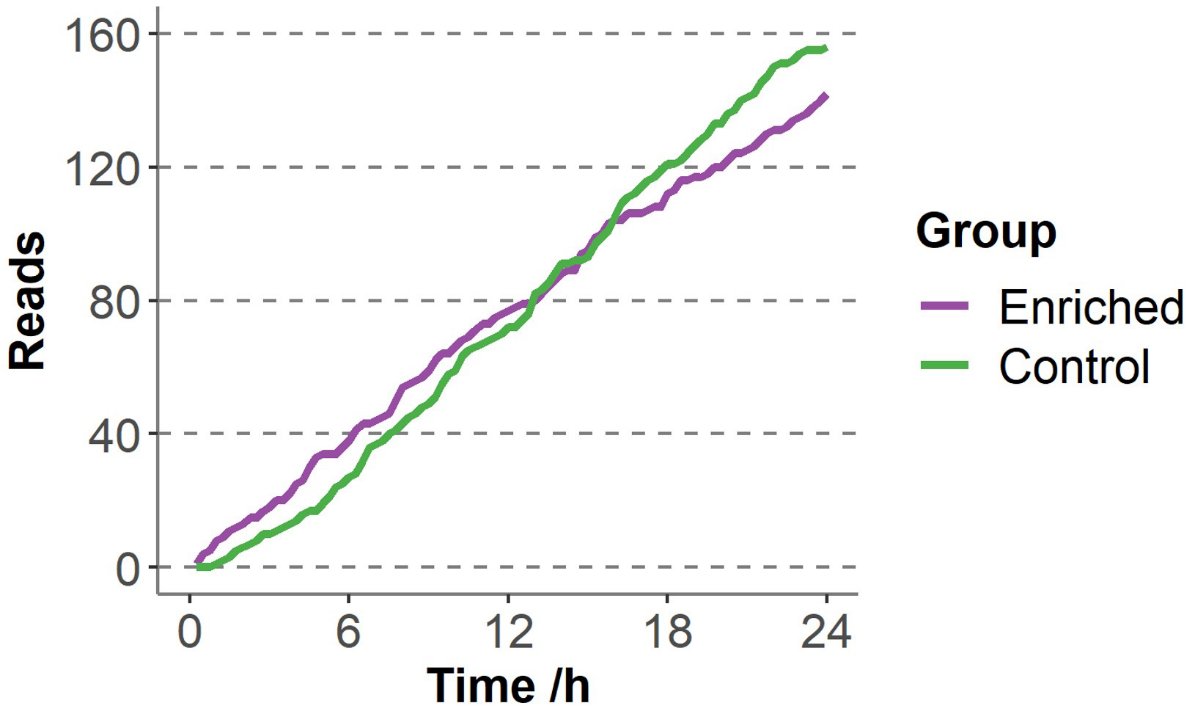


**Figure S2. The HAdV reads in the enriched group (purple) and control group (green) changed over 24 h for the sequencing of sample M9 with LNAS workflow.**
